# Open PHACTS computational protocols for *in silico* target validation of cellular phenotypic screens: knowing the knowns†
†The authors declare no competing interests.
‡
‡Electronic supplementary information (ESI) available: Pipeline Pilot protocols, xls file with the output of the Pipeline Pilot protocols, KNIME workflows, and supplementary figures showing the Pipeline Pilot protocols. See DOI: 10.1039/c6md00065g



**DOI:** 10.1039/c6md00065g

**Published:** 2016-05-11

**Authors:** D. Digles, B. Zdrazil, J.-M. Neefs, H. Van Vlijmen, C. Herhaus, A. Caracoti, J. Brea, B. Roibás, M. I. Loza, N. Queralt-Rosinach, L. I. Furlong, A. Gaulton, L. Bartek, S. Senger, C. Chichester, O. Engkvist, C. T. Evelo, N. I. Franklin, D. Marren, G. F. Ecker, E. Jacoby

**Affiliations:** a Department of Pharmaceutical Chemistry , University of Vienna , Pharmacoinformatics Research Group , Althanstraße 14 , 1090 Wien , Austria . Email: daniela.digles@univie.ac.at; b Janssen Research & Development , Turnhoutseweg 30 , B-2340 Beerse , Belgium . Email: ejacoby@its.jnj.com; c Merck KGaA, Merck Serono R&D , Computational Chemistry , Frankfurter Straße 250 , 64293 Darmstadt , Germany; d BIOVIA , a Dassault Systèmes brand , 334 Cambridge Science Park , Cambridge CB4 0WN , UK; e Grupo BioFarma-USEF , Departamento de Farmacología , Facultad de Farmacia , Campus Universitario Sur s/n , 15782 Santiago de Compostela , Spain; f Research Programme on Biomedical Informatics (GRIB) , Hospital del Mar Medical Research Institute (IMIM) , Department of Experimental and Health Sciences , Universitat Pompeu Fabra , C/Dr Aiguader 88 , E-08003 Barcelona , Spain; g European Molecular Biology Laboratory , European Bioinformatics Institute (EMBL-EBI) , Wellcome Genome Campus , Hinxton , Cambridge CB10 1SD , UK; h GlaxoSmithKline , Medicines Research Centre , Stevenage SG1 2NY , UK; i Swiss Institute of Bioinformatics , CALIPHO Group , CMU Rue Michel-Servet 1 , 1211 Geneva 4 , Switzerland; j Nestlé Institute of Health Sciences SA , EPFL Innovation Park, Bâtiment H , 1015 Lausanne , Switzerland; k Chemistry Innovation Centre , Discovery Sciences , AstraZeneca R&D Gothenburg , SE-431 83 Mölndal , Sweden; l Department of Bioinformatics – BiGCaT , P.O. Box 616 , UNS50 Box19 , NL-6200MD Maastricht , The Netherlands; m Open Innovation Drug Discovery , Discovery Chemistry Eli Lilly and Company , Lilly Corporate Center , DC 1920 , Indianapolis , IN 46285 , USA; n Eli Lilly and Company Ltd. , Lilly Research Centre , Erl Wood Manor, Sunninghill Road , Windlesham , Surrey GU20 6PH , England , UK

## Abstract

Phenotypic screening is in a renaissance phase and is expected by many academic and industry leaders to accelerate the discovery of new drugs for new biology. Given that phenotypic screening is per definition target agnostic, the emphasis of *in silico* and *in vitro* follow-up work is on the exploration of possible molecular mechanisms and efficacy targets underlying the biological processes interrogated by the phenotypic screening experiments. Herein, we present six exemplar computational protocols for the interpretation of cellular phenotypic screens based on the integration of compound, target, pathway, and disease data established by the IMI Open PHACTS project. The protocols annotate phenotypic hit lists and allow follow-up experiments and mechanistic conclusions. The annotations included are from ChEMBL, ChEBI, GO, WikiPathways and DisGeNET. Also provided are protocols which select from the IUPHAR/BPS Guide to PHARMACOLOGY interaction file selective compounds to probe potential targets and a correlation robot which systematically aims to identify an overlap of active compounds in both the phenotypic as well as any kinase assay. The protocols are applied to a phenotypic pre-lamin A/C splicing assay selected from the ChEMBL database to illustrate the process. The computational protocols make use of the Open PHACTS API and data and are built within the Pipeline Pilot and KNIME workflow tools.

## Abstract 2

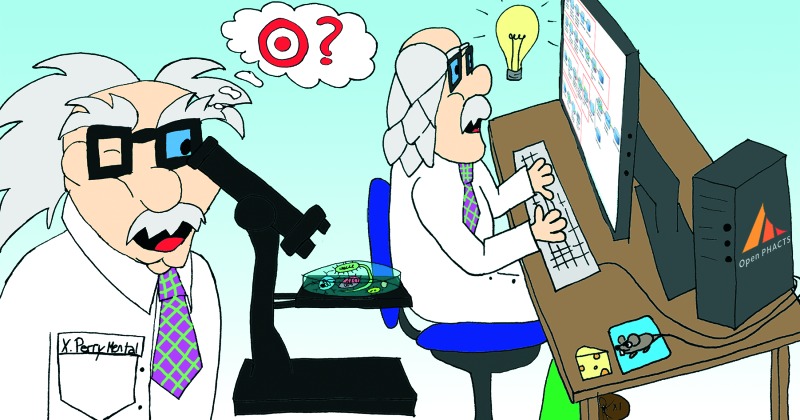
Six computational protocols to annotate phenotypic screens.

## Introduction

Even though the discussion is still ongoing whether or not phenotypic screening was historically more productive for the discovery of first in class drugs than target-directed screening, and whether it continues to do so, it is clear that phenotypic screening opens new avenues to investigate new cellular biology.^1–4^ In the 1970s, phenotypic screening on physiological whole animal or organ testing with a limited number of compounds was very popular and successful. Drug hunters like Paul Janssen or James Black tested pharmacologically rich compounds systematically on a broad panel of such phenotypic assays across a spectrum of therapeutic areas and discovered breakthrough medicines like antipsychotics, beta-blockers, or anti-ulceratives.^5^ Also, phenotypic screening analysis of approved drugs can generate new insights. Recently, Lee *et al.* screened a large collection of approved drugs in phenotypic assays including models for osteoporosis, diabetes and cancer, identifying novel activities for several known compounds.^6^

**Fig. 1:**
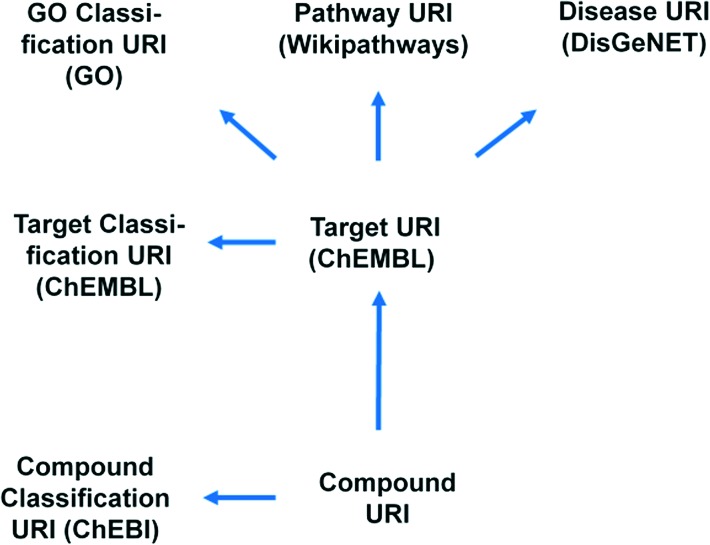
Outline of data showing the links between the different key identifiers (URI, uniform resource identifiers) assessed for annotation of compounds and targets in the provided computational protocols. The data provenance is shown in brackets.

With the fantastic progress in molecular and cellular biology, cell-based phenotypic screening in primary or engineered cell-lines constitutes a promising avenue. New biology like for instance alternative splicing or translational read through becomes experimentally accessible using MTS/HTS approaches. The experiments deliver potentially potent and specific compounds for which it can be interesting to elucidate and validate the molecular mechanism. Next to experimental target validation including chemogenomics pull-down and knock-in/out experiments, the *in silico* assessment of the hit lists constitutes a key step.^7–11^ This analysis requires a high level of data integration in order for it to be complete and seamless. Such integration was recently achieved by the IMI Open PHACTS project^12^ resulting in the Open PHACTS Discovery Platform (; www.openphacts.org).^13^ The Open PHACTS project uses semantic web technology for drug discovery by integrating relevant concept spaces of compound–target–pathway and disease (see Fig. 1 for concepts/URIs used in this work). This enables, as we will show herein, insightful interpretation of the phenotypic screening results to sustain target validation based on hitherto established drug discovery knowledge.

**Fig. 2:**
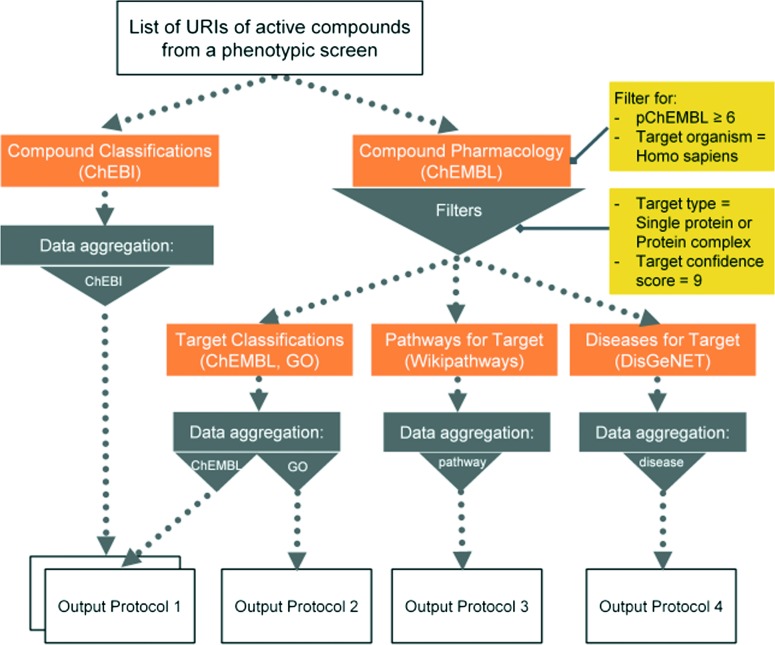
Schematic overview of protocols 1 to 4.

**Fig. 3:**
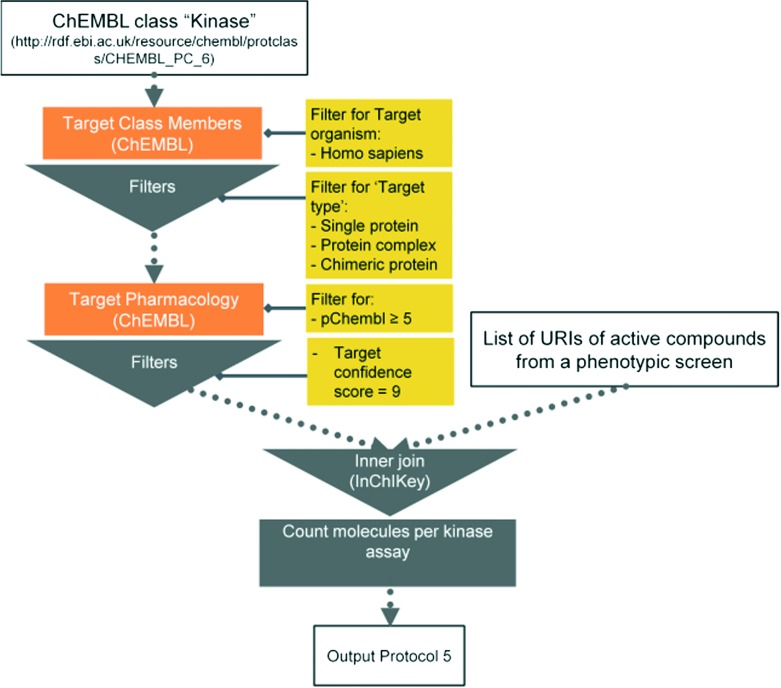
Schematic overview of protocol 5.

**Fig. 4:**
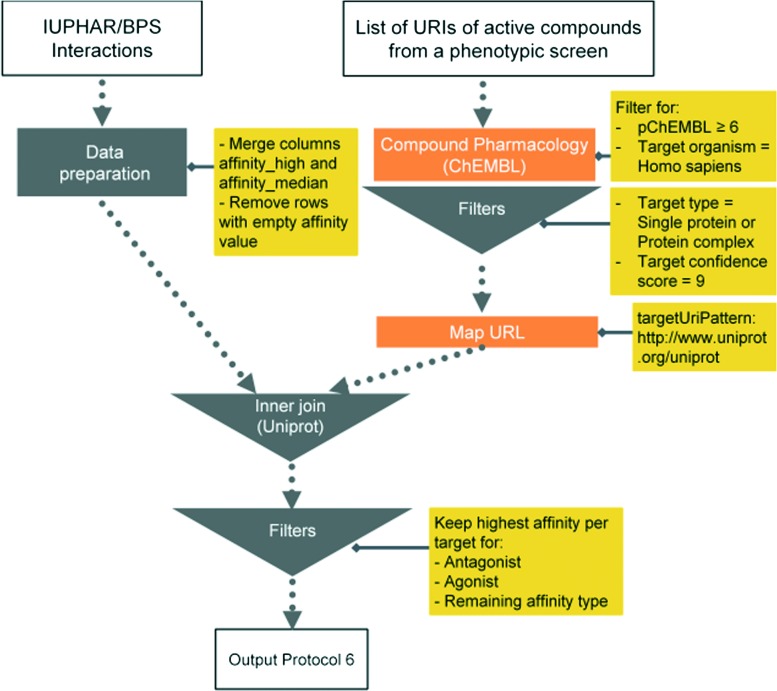
Schematic overview of protocol 6.

Here we present six protocols, which could be useful to annotate the results of a phenotypic screening experiment. Protocol 1 retrieves known classifications for compounds of interest. Protocols 1 to 4 retrieve targets, which these compounds have recorded bioactivity values for, and subsequently retrieves additional data for these targets (ChEMBL classification, GO terms, pathways, and diseases, respectively). These protocols are depicted in Fig. 2 and the implementation of protocol 1 in Pipeline Pilot is shown in Fig. S1.‡ Protocol 5 retrieves all kinases and reported bioactivity values available in the ChEMBL database and returns an overlap with the compounds from the phenotypic screening (Fig. 3 and Fig. S2‡). A possibility to join the data retrieved from the Open PHACTS Discovery Platform with external data is shown in protocol 6 (Fig. 4 and Fig. S3‡).

## Experimental

### Software

Workflows were generated first with Pipeline Pilot from BIOVIA,^14^ and were then adapted for KNIME.^15^ Pipeline Pilot protocols were created in version 9.2 using version 2.0 of the Open PHACTS component collection, which was downloaded from the BIOVIA ScienceCloud Exchange.

KNIME version 2.12.1 with installed JSON (KNIME Labs Extensions) and REST nodes (KNIME Community Contributions provided by Cenix BioScience) was used to create the workflows. Open PHACTS KNIME nodes (org.openphacts.utils.json_1.1.0) were retrieved from the github repository (https://github.com/openphacts/OPS-Knime).

### Execution of API calls

An overview of API calls used in this study is provided in Table 1. Documentation of the Open PHACTS API^13^ is available at ; http://dev.openphacts.org/.

**Table 1:** API calls and input, output and aggregation parameters

Protocol	Used API calls	Input	Output	Aggregation
1-2 ChEMBL, GO	Compound Pharmacology: List,	List of Compound URIs	Cache 1: CompoundID and URICompound, canonical smiles	Join cache 3 and 2 based on target name; Join cache 1 on URICompound
	Target Classifications		Cache 2: URI compound, URI assay, pChembl, TargetName, URITarget	Merge and group on classification
			Cache 3: Target Name, URIClassification, Classification	
1 Chebi	Compound Classifications	List of Compound URIs	Cache 1: CompoundID and URICompound, canonical smiles	Join cache 2 and 1 based on URICompound.
			Cache 2: URI compound, URI Chebi, ChebiDescription	Merge and group on ChebiDescription
3 Pathways	Compound Pharmacology: List,	List of Compound URI	Cache 1: CompoundID and URICompound, canonical smiles	Join cache 3 and 2 based on target name; Join cache 1 on URICompound
	Pathways for Target: List		Cache 2: URI compound, URI assay, pChembl, TargetName, URITarget	Merge and group on PathwayName
			Cache 3 : Target Name, URIPathway, PathwayID, PathwayName	
4 Disease	Compound Pharmacology: List,	List of Compound URIs	Cache 1 : CompoundID and URICompound, canonical smiles	Join cache 3 and 2 based on target name; Join cache 1 on URICompound
	Diseases for Target: List		Cache 2 : URI compound, URI assay, pChembl, TargetName, URITarget	Merge and group on DiseaseName
			Cache 3 : Target Name, URIDisease, DiseaseName	To limit the runtime of the protocol merging is done directly on the data stream of Cache 3.
5 Correlation Robot	Target Class Member: List,	Use CHEMBL_PC_6 kinase family key to launch query	Cache 1 : List of 455 human kinases for which ChEMBL holds data	Join Cache 2 based on INCHIKEY to each assay from Cache 1 data stream
	Target Pharmacology: List		Cache 2 : Lamin A/C splicing assay data	
6 GtoPdb Box	Compound Pharmacology: List,	List of Compound URIs. Cache 1 : Read GtoPdb interaction file	Cache 2 Keep URITarget and URIUniprot and extract UniprotID from URIUniprot	Join cache 1 GtoPdb interaction file to cache 2 based UniprotID. Merge by ligand name
	Map URL			

#### Pipeline Pilot

The Open PHACTS component collection provides one component per API call to facilitate the building of complex protocols. All components in the collection share the same logic inside, with the HTTP connector component performing a GET operation on the appropriate API endpoint. The output hierarchical data is then manipulated depending on user-defined parameters to extract only the desired parts of the hierarchy which are either flattened using the flatten hierarchy component or output as hierarchical data records that can be manipulated further.

#### KNIME

API calls were generated using the OPS_Swagger node (with https://raw.githubusercontent.com/openphacts/OPS_LinkedDataApi/1.5.0/api-config-files/swagger.json as definition for the calls). A string replacer node was used to update the calls to the latest public version (from 1.5 to 2.0). Data was retrieved using the GET Resource node and adapted for a tabular format with the String to JSON and JSON Path nodes.

### Data sources

Calls to the Open PHACTS Discovery Platform were made with the API version 2.0 (https://dev.openphacts.org/docs/2.0). Data collected in this study, which was accessed *via* the Open PHACTS Discovery Platform, includes ChEMBL^16,17^ version 20, ChEBI^18^ release 125, Gene Ontology (GO)^19,20^ annotations (accessed Feb. 2015), WikiPathways^21^ v20151118, and DisGeNET^22,23^ version 2.1.0.

The pre-lamin A/C splicing assay data was selected from ChEMBL *via* the Open PHACTS Discovery Platform with the ChEMBL1293235 target ID (target pharmacology: list call) and subsequent filtering for the CHEMBL1614310 assay ID.

Protocol 6 includes interaction data from the IUPHAR/BPS Guide to PHARMACOLOGY (GtoPdb)^24^ downloaded from ; http://guidetopharmacology.org/DATA/interactions.csv (accessed Oct. 2015).

## Results and discussion

### Protocol design

#### Protocol 1 – ChEBI/ChEMBL annotation and classification

One of the first questions a medicinal chemist is likely to ask when receiving a list of compounds from a phenotypic screen is related to the target classes that the molecules can be active against. Are there kinase inhibitors or GPCR ligands in the hit list and at which potency level? The known SAR of a given compound is obtained by using the *‘Compound Pharmacology: List’* API call. Then, two more API calls are needed for the target and compound annotations. The *‘Target Classification’* API call enables to retrieve the ChEMBL classification for the protein targets of interest by introducing their URIs as query input parameter. The *‘Compound Classification’* API call enables to retrieve the ChEBI annotation for the given compounds by introducing their compound URIs as input. For results interpretation, applying simple aggregation statistics on the number of individual assay activities, individual targets, or directly compounds allow to assess the relative relevance of the findings. Finally, output reports are generated at both the individual compound–target–activity level and at the aggregated level (*e.g.* by grouping according to the ChEMBL ‘Protein Classification’) (Fig. 2). The ChEBI annotation provides a first overview on the pharmaceutical and chemical classes covered. All available ChEBI annotation types are retrieved in the protocols, but especially the function annotation “has role”, and the chemical class of the molecule “Type” will be of interest here. This compound annotation facilitates the communication between chemists and biologists by providing a standardized language.

#### Protocol 2 – GO annotation

The Gene Ontology (GO) allows the annotation of gene products with molecular function, biological process, and cellular component information. The cellular component information captures the localization of the gene product in the cell, which can be a part of the cell (*e.g.* the plasma membrane) or a more specific component such as a protein complex. The molecular function describes the activity of the gene product at a molecular level (*e.g.* catalytic activity), while a biological process is a series of events that can be composed of several molecular functions.

In the Open PHACTS Discovery Platform, this information is accessible using again the ‘*Target Classification*’ API call, by inputting the protein target URIs in order to retrieve the GO classification trees. This information is highly complementary to the target annotation obtained in protocol 1. The information on the cellular component can be used to localize the site of action of the compounds provided that the underlying assay data is at the cellular level and not a cell-free biochemical format. The biological process and molecular mechanism information is essential for the assessment of the activities. Again, simple aggregation statistics at the level of individual assay activities, individual targets, or directly compounds allow an assessment of the relevance of the information. Observing, for instance, multiple highly potent compounds hitting different targets pointing to the same molecular process builds confidence of the relevance of this process for the observed phenotype. Given the richness of the GO terminology, its interpretation requires broad knowledge of general molecular and cellular biology and disease biology in order to assess the relevance.

#### Protocol 3 – WikiPathways annotation

Are multiple protein nodes of the same pathway hit by different compounds? This is a question of interest to build confidence that the particular pathway is of relevance for the observed phenotype. Also, it increases the meaningfulness of the target findings, if they all contribute to the same phenotypic outcome through the same process.

Extending the biological knowledge of a hit list is possible using the WikiPathways annotation. Like in protocol 1 and 2, the workflow first retrieves the (poly)pharmacology data for the given compounds, resulting in a list of targets. The use of the ‘*Pathways for Targets List*’ API call, which yields to the associated WikiPathways URIs and Names for an inputted protein target URI list, gives a pathway-based summarizing view of the bioactivities.

#### Protocol 4 – links to diseases and possible side effects – DisGeNET annotation

Knowledge about the diseases and side effects potentially associated to the compounds through the target link is of interest in order to either prioritize hits with potential synergistic effects or to deprioritize hits associated with adverse side effects. Also, knowledge about a disease with strong mechanistic overlap is informative. DisGeNET, a database on human diseases and their genes, is available through the Open PHACTS Discovery Platform. DisGeNET is one of the most comprehensive resources on gene-disease associations collected and integrated from a variety of authoritative sources on human genetics and the scientific literature, which covers the whole spectrum of human diseases. The disease annotations of the targets are retrieved from the Open PHACTS Discovery Platform *via* the API call ‘*Diseases for Target*: *List*’. The corresponding workflow is built in a similar manner as protocols 1–3, *i.e.* combining data calls and joining operations followed by computation of aggregation statistics.

#### Protocol 5 – correlation of the phenotypic and bio-chemical screening data

Working with phenotypic screening data, we observe that many hit lists contain a significant number of kinase inhibitors (E. Jacoby, unpublished results). This might be the result of the kinase focus of the compound libraries achieved in the last decade, but might also reflect the dominant role that kinases use to play in the modulation and regulation of signalling pathways.^25^ Additionally, kinase inhibitors were shown to retrieve active hits in phenotypic screening assays (NCI-60 panel and angiogenesis).^26^ One obvious question is therefore to identify potential correlations between the known kinase assays and the phenotypic assay of interest. Identified kinases might play a relevant role in the biological process interrogated by the phenotypic assay.

With the integration achieved in the Open PHACTS Discovery Platform, it is directly feasible to retrieve all kinases from the ChEMBL classification tree (‘*Target Class Member: List*’ call) as well as the connected pharmacological data (‘*Target Pharmacology: List*’ call). Alternatively, the ‘*Target Class Pharmacology: List*’ call can be used directly. We opt here to apply the most simple correlation type by analysing the number of hits in common in the assays to be compared. The assay correlation robot might obviously be applied to other target families or to the entire pharmacological space. This analysis is complementary to the analysis provided in protocol 1.

#### Protocol 6 – compound tool box to validate/devalidate identified potential targets of protocol 1 based on GtoPdb

GtoPdb provides a list of studied and validated compound interactions from scientific literature. Therefore, compounds which include probe compounds from the Structural Genomics Consortium (SGC, http://www.thesgc.org) and the Molecular Libraries and Imaging Program (MLP, ; http://mli.nih.gov/mli) can be used as potent and specific tools to validate/devalidate a potential target in a complementary manner to CRISPR-Cas9 (ref. 27) technology. The GtoPdb interaction file, is not yet integrated in the Open PHACTS Discovery Platform. However, due to the adoption of common protein identifiers (UniProt accessions) between resources, it is possible to use the ‘*Map URL*’ API call to retrieve a UniProt URI for each target based on the protein target URIs and join these with the GtoPdb interaction file containing compound–target interactions. In this way it is possible to get for each protein target of interest an associated compound list for testing.

This application demonstrates the flexibility by combining the Open PHACTS API and data workflow tools to integrate additional data sources. The protocol was designed in a manner to distinguish between agonist, antagonist, and other interaction types and to keep the most potent compound for each category for each target.

#### Workflow designs in Pipeline Pilot and KNIME

Given the technical differences and particularities of the Pipeline Pilot and KNIME workflow tools there are a number of differences in the overall architecture of the resulting workflows. Table 1 summarizes the used API calls and input, output and aggregation parameters as used in Pipeline Pilot implementation. Pipeline Pilot protocols are available from the BIOVIA ScienceCloud Exchange (; https://exchange.sciencecloud.com, search for keyword ‘openphacts’). The KNIME workflows can be downloaded from myExperiment,^28^ a collaborative environment for publishing workflows (; http://www.myexperiment.org/packs/707.html).

The Pipeline Pilot implementation makes full usage of the cache functionality which allows to store data using the API into data caches and then to join and aggregate it within a separate data stream. The design of the data pipelining protocols is made in a manner that at each step selected output data is cached and only the required input URIs are forward propagated to the next API calling node (see Fig. S1–S3‡). The outputs are then joined and grouped in a sequential manner to produce the desired information. This principle illustrates a key advantage for data mining. In this approach data is obtained collectively and the user sorts out and selects the desired information afterwards. Protocols 1 to 4 were combined into a single workflow in KNIME to reduce calculation time for redundant steps.

#### Application to correctors for lamin A splicing assay

In order to demonstrate the capabilities of the protocols, we applied them to the pre-lamin A/C splicing assay from the PubChem BioAssay database (AID:1487 (ref. 29)). This Pubchem assay has the title ‘PUBCHEM_BIOASSAY: Validation of Assay for Modulators of Lamin A Splicing’ in ChEMBL (Assay ID CHEMBL1614310) and lists 280 bioactivities for which 85 different compounds have pChEMBL values ≥5 (containing both mildly active and very active compounds), which are further analysed hereafter. The assay measures expression of correctly spliced protein and was generated within the NIH Molecular Libraries Probe Production Network.^30^ It aims to identify splicing correctors against the Hutchinson–Gilford progeria syndrome (HGPS). HGPS is a paediatric premature aging disease caused by a spontaneous mutation in the lamin A/C (LMNA) gene. The mutation activates a cryptic splice site in the LMNA pre-mRNA which results in production of a pre-lamin A protein that cannot be processed properly. The mutant protein accumulates in the nucleus and negatively affects numerous cellular functions.

The resulting data for this application example from the Pipeline Pilot protocols are provided in the ESI.‡ Investigation of the target classifications (protocol 1) show that 47 kinase activities are observed based on 8 compounds on 27 targets. Interesting are the CGMC kinases DYRK1A and GSK3B and the MAP Kinases p38 α and β, c-Jun2 and 3 and ERK2. DYRK1A inhibitors are reported in the literature to modulate alternative pre-mRNA splicing of model gene transcripts in cells with submicromolar potencies.^31^ For the family A GPCR, 56 activities are observed based on 14 compounds on 19 targets. Most prominent are the monoamine receptor activities. 27 epigenetic regulator activities are observed based on 17 compounds on five targets.

Regarding GO component, 186 terms are found; 13 compounds are linked to the spliceosomal complex *via* the heterogeneous nuclear ribonucleoprotein A1 and the survival motor neuron protein. For GO process, 1287 terms are found. Multiple compounds are linked to various DNA related processes *via* the Bloom syndrome protein, while 13 compounds are linked to spliceosomal complex assembly. For GO function, 340 terms are found. The kinase assay correlation robot supports the hint to kinases and points to the MAP kinase ERK2 assay CHEMBL1613808 which has eight compounds in common. The underlying pathway is the MAPK signalling pathway, which is found in protocol 3 for 12 targets.

In general, 306 pathways are identified, with ‘GPCR downstream signaling’ and ‘GPCR ligand binding’ showing the highest count of identified targets (19), and ‘FAS pathway and Stress induction of HSP regulation’ and ‘Integrated Pancreatic Cancer Pathway’ showing the highest count of active molecules (37).

The DisGeNET annotation (protocol 4) provides links to 3631 diseases and side-effects; 89 of them have more than 20 potential efficacy targets links. Various neoplasms and cancers are prominent given the link *via* kinases. Spinal muscular atrophy is linked by 13 hits *via* the survival motor protein link. It will require further disease biology expertise to recognize relevant links to the observed phenotype.

ChEBI terms (protocol 1) associated with at least five compounds include five metabolites and nine antineoplastic agents among which fluorouracil, camptothecin and rotenone are listed. Rotenone is discussed in the literature to modulate splicing of several genes, *e.g.* alternative splicing of the X-linked NDUFB11 gene of the respiratory chain complex I.^32^

The analysis from protocol 6 suggests testing of 79 compounds in the phenotypic screening assay. Very prominent are monoamine receptor ligands and kinase inhibitors.

After having competed the *in silico* annotation, an obvious questions aims towards the modus operandi for follow-up experiments and drug discovery. A first obvious experiment is to test the tool compounds retrieved from the GtoPdb database in the phenotypic assay to verify if they produce the desired phenotype. Obviously, *in vitro* target validation through, for instance, CRISPR/cas9 experiments would complete the experimental target validation. In a similar perspective, ChEMBL biological annotations point to targets which enable such testing. The usage of the results from the correlation robot opens the possibility to substitute the phenotypic assay for the mechanistic target based assay for as the primary screening or optimization assay. This might allow for instance for higher throughput in screening. Given the low number of common hits found between the MAP kinase ERK2 assay and the pre-lamin phenotypic assay, we would recommend to test further ERK2 reference compounds in the phenotypic assay before taking a decision. A key difficulty relies in making sense of the GO, WikiPathways and DisGeNET annotations. Given that each putative target pulls potentially a multitude of these annotation categories, a clear navigation strategy is missing. One possible way forward could be the analysis of similarities between the annotations. Further work and domain expertise is needed to achieve this. Practically, the hint that 13 compounds are linked to the biology of the spliceosome complex increases the attractiveness of these compounds for follow-up chemistry lead optimization. Conversely, the link to activity on the Bloom syndrome protein flags a different set of compounds as potentially problematic, given the link to genomic instability of this protein.

## Conclusions

Knowing the knowns about a phenotypic screening hit list is a first essential step in the analysis of every phenotypic screening project and contributes to the validation of the potential efficacy targets associated to the hits.^33^ Collected knowledge might help to decide whether the research team should optimize hit compounds based on a phenotypic readout or pursue with mechanistic target based assays. The interpretation of the provided information requires broad knowledge of general molecular and cellular biology, as well as disease biology in order to assess their relevance. Importantly, this data-driven collected knowledge also guides the assessment of potential off-biology, including pharmacological and toxicological side-effects early in the projects. Herein presented computational protocols focus on cellular phenotypic screening data where the link to molecular mechanisms is possible.

A limitation of the here presented analysis could be a bias in the available data in the public domain databases used. In Open PHACTS we mainly use one data source for each of the data types (*e.g.* ChEMBL for bioactivities, DisGeNET for Diseases, WikiPathways for pathways). While most of these sources combine data from several places, this could lead to a bias in the data. The illustrated analyses will benefit from the inclusion of data beyond the Open PHACTS Discovery Platform, as for instance commercial data sources (like GOStar from GVKbio^34^) or patent extracted data (like SureChEMBL^35^). Additionally, API calls to other available data sources (*e.g.* the Entrez Utilities API Eutils) could be integrated into the protocols, to increase the coverage of the returned data. The corporate internal SAR data stores with massive amounts of fulldeck screening data will not only enable to include proprietary compounds into the analysis, but also to have access to more complete SAR data matrices. This will be of benefit especially for correlation analyses. The inclusion of negative screening results becomes equally possible with the corporate *in house* data. Negative data is of particular value given that the identified target proteins for phenotypic negatives cannot be dominant phenotypic targets. This is a very important point. In a typical *in silico* deconvolution effort, many active compounds will point to promiscuous targets, *e.g.* biogenic amine GPCRs,^36^ or point to generic pathways, *e.g.* ‘GPCR downstream signaling’. However, when putting these activities into context and showing that a similar or greater percentage of phenotypically inactive compounds are also hitting these targets, they can be removed from the list of potentially interesting efficacy targets. At this stage a rigorous statistical analysis will enable to distinguish a real signal from noise and help to interpret the results. It will thus be relevant to store the inactives of a phenotypic screen to enable such analysis.

Further restrictions on the suggested targets might appear from the inclusion of gene expression data. This is already feasible with the Open PHACTS Discovery Platform. The inclusion of protein complex information offers an equally interesting extension possibility of the pathway analysis protocol 3. A relevant question is: are multiple members of a given functional complex hit by different compounds? This aspect can be in part addressed by the GO cellular component annotation. Also, a combination of pathway and disease information could be worthwhile, to investigate the overlap of both.

Extending beyond the known knowns is possible by applying predictive chemogenomics SAR models which are currently being developed in academia and industry.^37–40^ Especially noteworthy are the predictive inference capabilities intrinsic to semantic approaches which allow integrating similarities among the data. Similarities between compounds, as well as similarities between proteins at the sequence level or even binding site level, can directly be coded in RDF. An extended version of the protocols, integrating experimental and predicted data would obviously top rank targets for which there is experimental evidence, and then, highlight the additional conclusions drawn from the predictions. The added value of the predictions is to potentially extend to the discovery of novel targets, not belonging to the known knowns.
