# Supplementary material for: Open PHACTS computational protocols for in silico target validation of cellular phenotypic screens: knowing the knowns
Source: Medchemcomm. 2016 May 11;7(6):1237–44. doi: 10.1039/c6md00065g (PMC5063042; doi:10.1039/c6md00065g)
Supplement: Supplementary file 1 [file MD-0183-C6MD00065G-s001.pdf]

Supplementary material

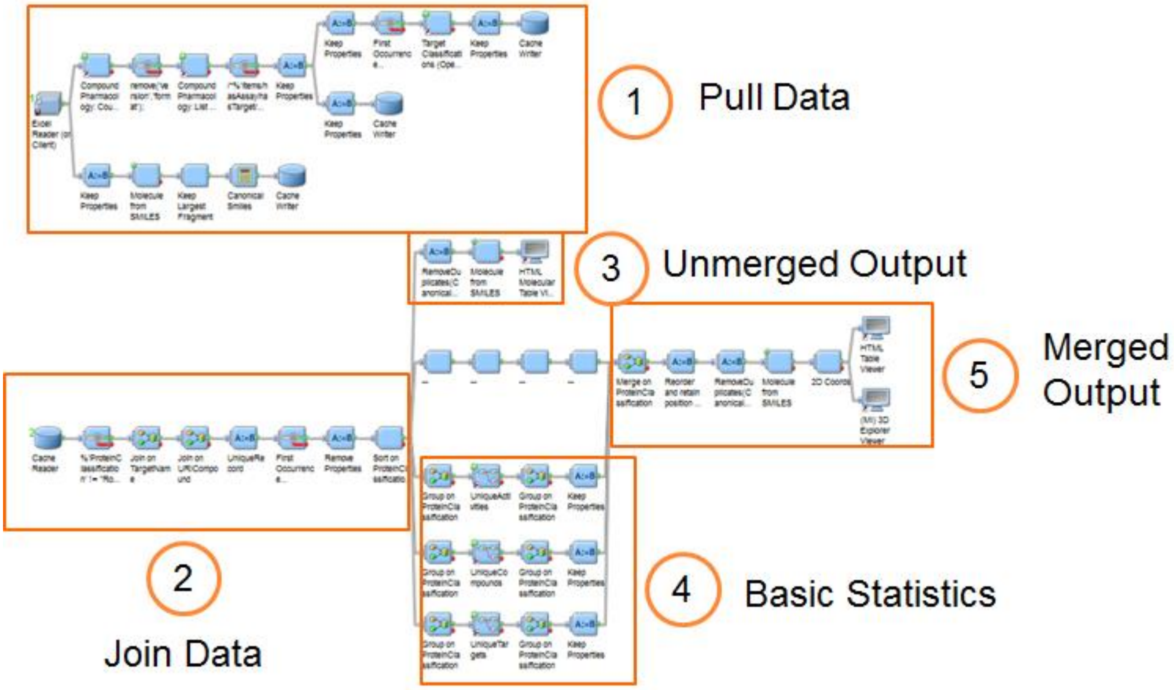

Supplementary Figure 1: Pipeline Pilot implementation of Protocol 1. The workflow consists of two data streams. In data stream 1, starting from a compound URI list, the data is retrieved using the Compound Pharmacology: List and Target Classification API calls. The target classification, the target and compound data are written respectively in the data caches 3, 2 and 1. In data stream two the data is in step 2 joined in reversed order starting from the target classification and joining on target URI and compound URIs. An unmerged output is produced in step 3. In step 4 basic aggregation statistics, and the unique activities, targets and compounds are computed. In step 4, the output is generated (merged by target classification).

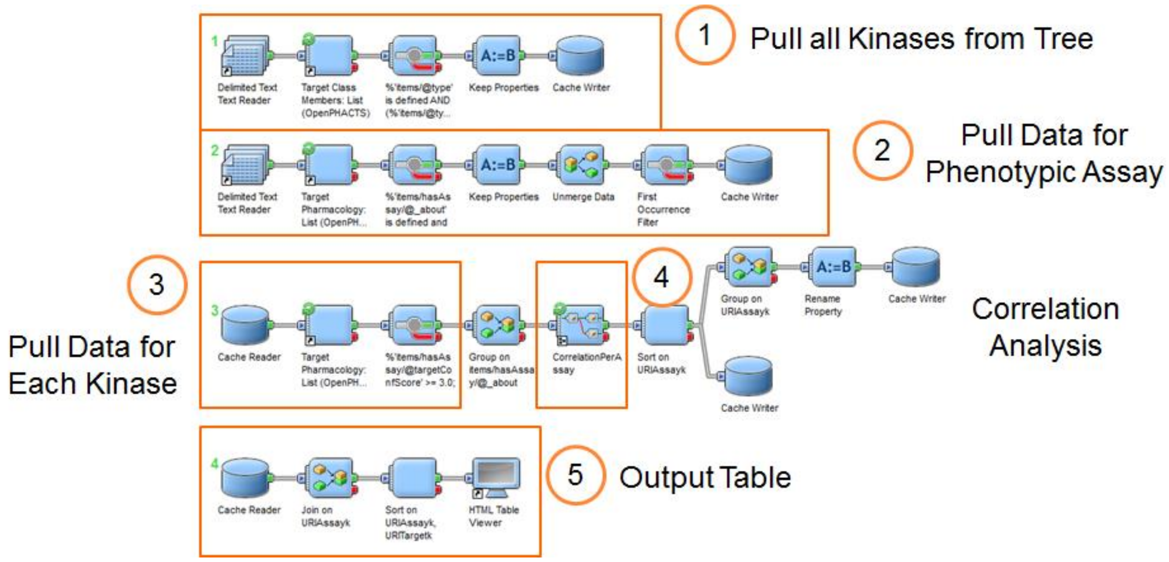

Supplementary Figure 2: Pipeline Pilot implementation of Protocol 5: correlation robot. In data stream 1 - step 1, all kinases are retrieved from the ChEMBL target classification tree

using the Target Class Members: List API call accessed with the kinase class ID; the list of kinases is written into cache 1. Data stream 2 – step 2, fetches the assay data for the pre-lamin A assay and writes it into cache 2. In data stream 3, the assay data for each of the kinases is retrieved using the Target Pharmacology: List API call. In step 4, using for each kinase a group by assay operation the correlation with the data from cache 2 is computed, using data joining on the common compounds. In data stream 4 – step 5 the final output table is generated.

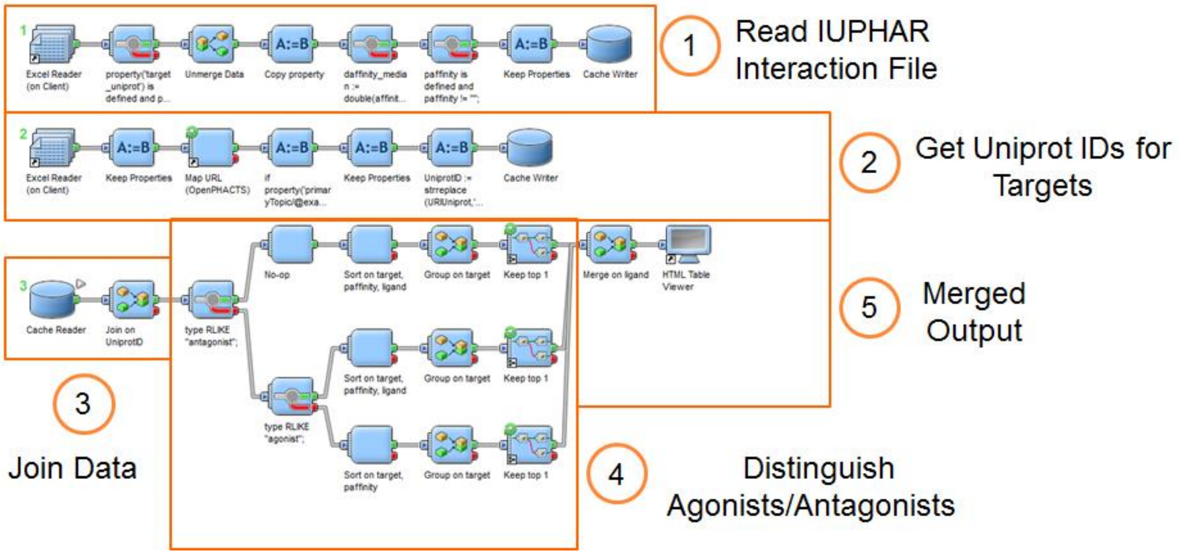

Supplementary Figure 3: Pipeline Pilot implementation of Protocol 6: GtoPdb. In data stream 1 - step 1, the IUPHAR interaction file is read into cache 1. Data stream 2 – step 2, extracts the UniProt Accession Numbers for each of the putative targets using the MAP URL API call; data is written into cache 2. Data stream 3 joins the data of the interaction file – cache 1 – with the actual list of putative relevant targets based on the UniProt IDs and separates sequentially the data –step 4 - for “antagonists”, “agonist” and “other” types of compounds. In step 5, a merged output based on the ligand names is generated.
